# A dataset on type specimens of hemipteran insects in China

**DOI:** 10.3897/BDJ.9.e64443

**Published:** 2021-06-29

**Authors:** Junjie Li, Huanhuan Liu, Yangxue Wu, Longqin Ye, Xiaolei Huang

**Affiliations:** 1 State Key Laboratory of Ecological Pest Control for Fujian and Taiwan Crops, College of Plant Protection, Fujian Agriculture and Forestry University, Fuzhou, China State Key Laboratory of Ecological Pest Control for Fujian and Taiwan Crops, College of Plant Protection, Fujian Agriculture and Forestry University Fuzhou China

**Keywords:** type specimen, Hemiptera, biodiversity, distribution, China

## Abstract

**Background:**

Type specimens are valuable resources for investigating and exploring biodiversity on Earth, which has high academic and conservation value. Hemipteran insects are one of the most important and diverse groups in Insecta and their type specimens have important reference value for many research fields. So far, the data on the type specimens of the Hemiptera in China have not been fully collated.

**New information:**

Through extensive literature review, we have constructed a dataset of type specimens for the new species of hemipteran insects in China published from 1950 to 2017, which includes the data such as collection date, specimen gender, preservation institution and geographical distribution. A total of 6,583 type specimen records were collected, covering 3,783 new species belonging to 1,299 genera and 88 families. This dataset can support the international community in conducting research on taxonomy, biodiversity, evolution and pest management.

## Introduction

Type specimens include name bearing specimens that have been designated by researchers when describing and publishing new species, such as holotypes and syntypes, which can be used as standard references for subsequent taxonomic investigation (Robinson 1975, Mutanen et al. 2015). The number of type specimens reflects not only the research status of taxonomy, but also the historical resource accumulation of a country or region (Yang 2013). The type specimens, including holotype, allotype, paratype and syntypes, are the most authentic and direct manifestations and physical records of various organisms in nature (He et al. 2019). In addition to being the basis and carrier for the establishment of new taxonomic categories (Haber 2012), they are also important references for carrying out research in biodiversity science, ecology and evolutionary biology and protecting biological resources (Cui et al. 2009, Bebber et al. 2012, Peterson 2014).

Hemiptera (Arthropoda: Insecta) is not only the largest hemimetabolous order in the Insecta (Li et al. 2017), but also one of the most important and diverse insect groups (Schuh and Slater 1995). They are widely distributed around the world, with about 103,590 species having been recorded (Stork 2018). Most of them are phytophagous, therefore, they include important pests in agriculture and forestry, such as aphids, scale insects, leafhoppers as well as planthoppers (Forero 2008, Guo and Yuan 2016, Li et al. 2019). The type specimens of hemipteran insects are important references for the study of taxonomy, systematics, biogeography and pest control. China has a vast territory and spans two zoogeographic regions, the Palaearctic Region and the Oriental Region (Chen et al. 2008, Lei et al. 2015). Due to its heterogeneous environment and diverse habitat types, China is one of the most biologically diverse countries in the world (Myers et al. 2000, Tang et al. 2006, Lu et al. 2018). Up to now, however, the data of type specimens of Chinese hemipteran insects have not been well organised. There is no comprehensive digital resource of hemipteran type specimens available for scientists. In view of the importance of insect type specimens in entomology and biodiversity research, we have constructed a type specimen dataset of the Chinese hemipteran insects in order to provide basic references for future studies.

## General description

### Purpose

The aim of this work is to compile the dataset of type specimens of the Hemiptera in China.

### Additional information

**Figure 1.**
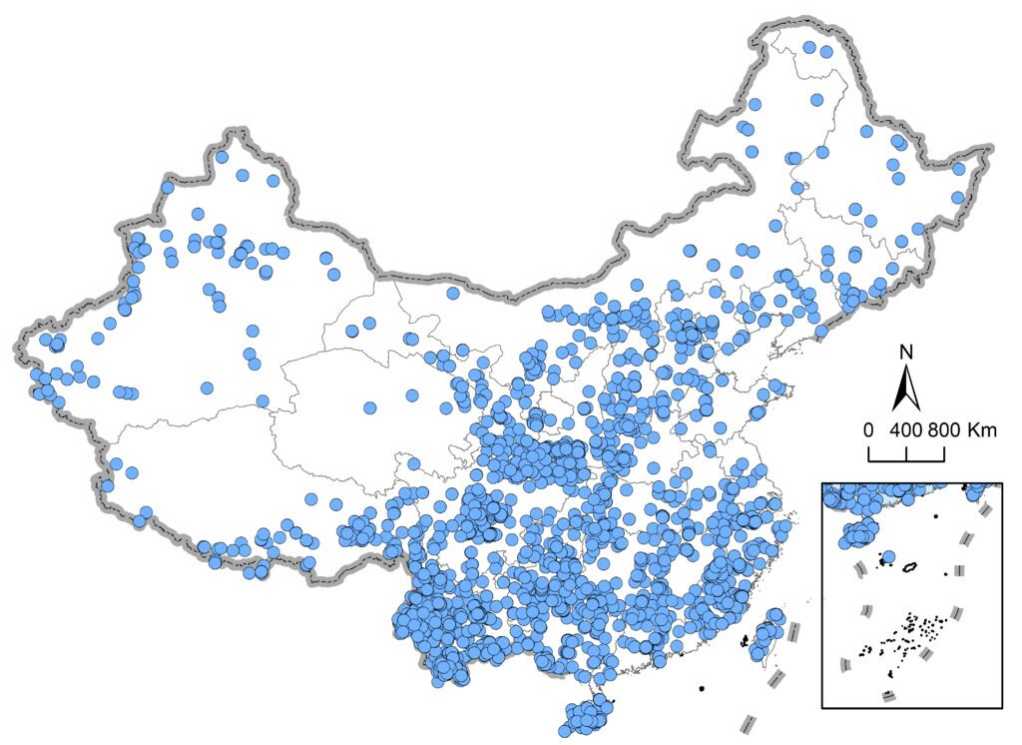
Geographical locations of the type specimens of Chinese hemipteran insects.

The collection date, specimen gender, preservation institution, geographical distribution and other related information of the holotypes, allotypes and paratypes for species of Hemiptera have been recorded in detail from various data sources, including scientific journals, serial publications, local chronicles, monographs and books. We have compiled almost all type specimen information for hemipteran insects published from 1950 to 2017 in China. The final dataset contains 6,583 records of 3,783 Hemiptera belonging to 1,299 genera and 88 families (Table 1) and covering a large number of areas (Fig. 1). A total of 418 authors participated in the description of the type specimens of Hemiptera and all type specimens are stored in 84 preservation institutions from 14 countries including China, the United Kingdom, Russia, the United States, Australia, Poland, France, Belgium, Japan, Austria, Germany, Netherlands, Singapore and India, among which all holotypes are stored in 66 preservation institutions in 10 countries. The holotype of most species (3,596 species, 99.39%) are preserved in China, with only 22 species (0.61%) stored in nine other countries, as shown in the Table 2.

This article provides a detailed description of the data source, structure and processing of the type specimen dataset of the Chinese hemipteran insects and presents the potential reuse value of this dataset. We are committed to making this dataset a dynamic one by following the principles of open science and constantly updating available new records. Through this work, we hope to promote further development of insect data collation and provide assistance to the research of biodiversity and entomology.

## Sampling methods

### Sampling description

**Data Sources**

The dataset mainly collected the type specimens of Chinese Hemiptera published by domestic and foreign scientists from 1950 to 2017. Our data sources consisted of two parts, one of which is mainly from the book series Catalogue of Insect Type Specimens Deposited in China (Cui et al. 2007, Cui et al. 2009, Bai et al. 2014). This series of books mainly records the data of insect type specimens produced in China published from 1950 to 2010 and the references mostly come from journals, chorographies, serial publications and monographs. Specifically, professional journals and chorographies mainly include the *Acta Entomologica Sinica, Entomotaxonomia, Zoological Systematics, Wuyi Science Journal, Zoological Research, Acta Zoologica Sinica, Scientia Silvae Sinicae, Journal of Beijing Forestry University, Entomological Journal of East China, Journal of South China Agricultural University, Sichuan Journal of Zoology, Journal of Ningxia Agricultural College, Journal of Northwest Forestry University, Acta Agriculturae Boreali-occidentalis Sinica, Journal of Northwest A & F University, Journal of Southwest Forestry College and the Journal of Southwest Agricultural University*. Serial publications mainly include the Fauna Sinica, Collected Papers of Entomological Research, the Insect Fauna in Henan Province and the Insects of Fujian Province. Monographs mainly include the Insects of the Hengduan Mountains and the Insects of the Three Gorge Reservoir Area of Yangtze River.

In addition, we also searched relevant literature published from 2000 to 2017 based on the Zoological Records in order to include recent species records and make the dataset more comprehensive. The search entry used was (new speci* or new tax*) and (Hemiptera or Homoptera) and (China). We retrieved 371 literature records, with information including the authors, title, abstract, publication date and journal information of each record. Based on these records, we obtained complete published article for subsequent specimen data collection. There were eight records about extinct species and one without a new species description, which was excluded from data collection.

**Data collection and processing**

We searched and collected the type specimen information of each new Hemiptera, including the species/subspecies name and its taxonomic status, the year of publication, as well as the gender, distribution, preservation institution and collection date of the holotype, allotype and paratype, respectively. In addition, each row of data was marked with its literature source and literature authors. In the original literature, several items, such as the species name, literature authors, preservation institution and collection date, were expressed in various ways. For example, some species names were with named person, while others were not. The literature authors might include both Chinese and foreign colleagues, and their names were written in different formats. Some names of preservation institutions were abbreviated, while others were with full names. Therefore, for the species names, we standardised them into the form of genus name plus species name. Literature authors were standardised as the last names followed by the initials of given names. The collection date was recorded as year, month and day. We used abbreviations for all preservation institutions for the sake of standardisation and their corresponding full names were shown in Table 3. Most literature only recorded the names of collection localities of the type specimens. In order to make the dataset more convenient for future users, we georeferenced the longitude and latitude of each distribution site using Google Maps. The coordinate precision is approximately 1,000 m. We recorded the few distribution records that provided coordinates. We translated the names of distribution sites from Chinese into English. For the literature sources, due to many species being published in Chinese, we compiled a separate ‘Literature’ column uniformly presented in English, but retained the original Chinese literature information in a ‘Original literature’ column.

### Quality control

After the completion of the original data collection, we checked all data records individually and standardised the format. In order to investigate whether the taxonomic information of each species/subspecies has changed, we checked the species/subspecies name and taxonomic status of each species/subspecies through the Catalogue of Life (http://www.catalogueoflife.org) and some other taxonomic websites for specific groups of Hemiptera, such as the Aphid Species File (http://aphid.speciesfile.org), the Systematic Database of the Scale Insects of the World (http://scalenet.info/catalogue), the Coreoidea Species File (http://Coreoidea.SpeciesFile.org) and the Lygaeoidea Species File (http://Lygaeoidea.speciesfile.org). If the species/subspecies name and its taxonomic status changed, we recorded them in corresponding fields of the dataset and the results showed that 11.9% of the species/subspecies names have changed since their original description. These changes are important records representing the taxonomic status of these species in different historical periods. In addition, we also checked and validated the geographical locations of the type specimens and their corresponding latitudes and longitudes in detail, based on original literature.

## Geographic coverage

### Description

China

### Coordinates

 and 3°51′N-53°33′N Latitude; and 73°33′E-135°05′E Longitude.

## Taxonomic coverage

### Description

Type specimens information for a total of 3,783 species of Hemiptera belonging to 1,299 genera and 88 families was collected.

## Temporal coverage

### Notes

Time range: 1950-2017

## Usage licence

### Usage licence

Creative Commons Public Domain Waiver (CC-Zero)

## Data resources

### Data package title

A dataset on type specimens of Hemipteran insects in China.

### Number of data sets

1

### Data set 1.

#### Data set name

A dataset on type specimens of Hemipteran insects in China.

#### Number of columns

30

#### Description

The final dataset is presented in Suppl. material 1, with the title of A dataset on type specimens of Hemipteran insects in China. At the same time, the dataset is also deposited in the DataOpen repository: http://doi.org/10.24899/do.202106001. The corresponding website is http://www.dataopen.info/home/datafile/index/id/210. Each row of the dataset represents the type specimen information of a species/subspecies, and if a species/subspecies contains multiple paratypes, it corresponds to multiple rows. The dataset contains 30 fields, as shown below:

**Data set 1.** 

Column label	Column description
ID	The unique number for each record.
Family	Family name of species/subspecies.
Family change	If the taxonomic status of a species/subspecies has changed in history, this indicates its current family name.
Genus	Genus name of species/subspecies.
Genus change	If the taxonomic status of a species/subspecies has changed in history, this indicates its current genus name.
Species/Subspecies name	The name of species/subspecies in a uniform format.
Species/Subspecies name change	If the name of a species/subspecies has changed in history, this indicates its current name.
Species/Subspecies names in the original literature	Species/Subspecies names recorded with various formats in original literature.
Published year	The year in which the species/subspecies was published.
Gender of holotype	The gender of the holotype used for species/subspecies description. This data item follows the original literature.
Distribution of holotype	The geographical location of the holotype.
Latitude of holotype	The latitude of the geographical location of the holotype.
Longitude of holotype	The Longitude of the geographical location of the holotype.
Collection time of holotype	Collection time of the holotype in a uniform format.
Preservation institution of holotype	The abbreviation for the preservation institution of the holotype.
Gender of allotype	The gender of the allotype used for species/subspecies description. This data item follows the original literature.
Distribution of allotype	The geographical location of the allotype.
Latitude of allotype	The latitude of the geographical location of the allotype.
Longitude of allotype	The longitude of the geographical location of the allotype.
Collection time of allotype	Collection time of the allotype in a uniform format.
Preservation institution of allotype	The abbreviation for the preservation institution of the allotype.
Gender of paratype	The gender of the paratype used for species/subspecies description. This data item follow the original literature.
Distribution of paratype	The geographical location of the paratype.
Latitude of paratype	The latitude of the geographical location of the paratype.
Longitude of paratype	The longitude of the geographical location of the paratype.
Collection time of paratype	Collection time of the paratype in a uniform format.
Preservation institution of paratype	The abbreviation for the preservation institution of the paratype.
Literature	The literature source of the species/subspecies, which is uniformly presented in English. If a journal name has been changed, its new name is reserved.
Original literature	Original literature information without modification.
Literature authors	The authors of the Literature.

## Supplementary Material

DADDEB5A-FA70-5123-8A09-13E6E13A5EF7 10.3897/BDJ.9.e64443.suppl1 Supplementary material 1 A dataset on type specimens of Hemipteran insects in China Data type A plain text table on the type specimens of Hemipteran insects in China File: oo_557255.txt https://binary.pensoft.net/file/557255 Junjie Li, Huanhuan Liu, Yangxue Wu, Longqin Ye, Xiaolei Huang

## Figures and Tables

**Table 1.** The diversity of Hemipteran insects included in this dataset.

Families	No. of species	Percentage of all species (%)	No. of genera	Percentage of all genera (%)
Cicadellidae	1120	29.61	256	19.71
Miridae	244	6.45	73	5.62
Aphididae	233	6.16	112	8.62
Psyllidae	221	5.84	33	2.54
Membracidae	179	4.73	41	3.16
Delphacidae	169	4.47	88	6.77
Reduviidae	154	4.07	66	5.08
Coreidae	128	3.38	56	4.31
Diaspididae	109	2.88	49	3.77
Pentatomidae	109	2.88	53	4.08
Lygaeidae	88	2.33	40	3.08
Triozidae	86	2.27	20	1.54
Cicadidae	75	1.98	34	2.62
Urostylidae	56	1.48	5	0.38
Aradidae	55	1.45	24	1.85
Pseudococcidae	53	1.4	32	2.46
Pemphigidae	46	1.22	20	1.54
Nabidae	42	1.11	11	0.85
Anthocoridae	37	0.98	6	0.46
Plataspidae	36	0.95	9	0.69
Lachnidae	35	0.93	8	0.62
Tingidae	34	0.9	21	1.62
Acanthosomatidae	29	0.77	7	0.54
Callaphididae	28	0.74	15	1.15
Aphrophoridae	22	0.58	5	0.38
Issidae	21	0.56	10	0.77
Veliidae	21	0.56	5	0.38
Drepanosiphidae	18	0.48	11	0.85
Achilidae	18	0.48	5	0.38
Aphalaridae	17	0.45	11	0.85
Chaitophoridae	17	0.45	3	0.23
Cercopidae	15	0.4	10	0.77
Derbidae	15	0.4	8	0.62
Cydnidae	14	0.37	8	0.62
Ricaniidae	14	0.37	7	0.54
Berytidae	13	0.34	5	0.38
Margarodidae	13	0.34	4	0.31
Hormaphididae	12	0.32	10	0.77
Tropiduchidae	12	0.32	7	0.54
Asterolecaniidae	10	0.26	5	0.38
Greenideidae	10	0.26	4	0.31
Flatidae	9	0.24	7	0.54
Coccidae	9	0.24	7	0.54
Kinnaridae	7	0.19	2	0.15
Dictyopharidae	7	0.19	5	0.38
Adelgidae	6	0.16	4	0.31
Cixiidae	6	0.16	5	0.38
Eurybrachidae	6	0.16	1	0.08
Machaerotidae	6	0.16	2	0.15
Piesmatidae	6	0.16	1	0.08
Aleyrodidae	5	0.13	5	0.38
Fulgoridae	5	0.13	3	0.23
Pyrrhocoridae	5	0.13	5	0.38
Kermesidae	5	0.13	2	0.15
Aphelocheiridae	4	0.11	1	0.08
Calophyidae	4	0.11	2	0.15
Ceratocombidae	4	0.11	1	0.08
Schizopteridae	4	0.11	4	0.31
Kerriidae	4	0.11	4	0.31
Velocipedidae	4	0.11	1	0.08
Eriococcidae	3	0.08	3	0.23
Gerridae	3	0.08	2	0.15
Lecaniodiaspididae	3	0.08	3	0.23
Meenoplidae	3	0.08	2	0.15
Thelaxidae	3	0.08	1	0.08
Rhyparochromidae	3	0.08	1	0.08
Nogodinidae	3	0.08	1	0.08
Lophopidae	2	0.05	2	0.15
Caliscelidae	2	0.05	1	0.08
Saldidae	2	0.05	2	0.15
Stenocephalidae	2	0.05	2	0.15
Scutelleridae	2	0.05	2	0.15
Eubranchidae	2	0.05	2	0.15
Rhophalidae	2	0.05	2	0.15
Notonectidae	1	0.03	1	0.08
Cerococcidae	1	0.03	1	0.08
Naucoridae	1	0.03	1	0.08
Anoeciidae	1	0.03	1	0.08
Leptopodidae	1	0.03	1	0.08
Isometopidae	1	0.03	1	0.08
Liviidae	1	0.03	1	0.08
Lyctocoridae	1	0.03	1	0.08
Mindaridae	1	0.03	1	0.08
Kuwaniidae	1	0.03	1	0.08
Aclerdidae	1	0.03	1	0.08
Tessaratomidae	1	0.03	1	0.08
Phloeomyzidae	1	0.03	1	0.08
Colobathristidae	1	0.03	1	0.08
Total	3783	100	1299	100

**Table 2.** The number of holotypes of Hemipteran species preserved by different preservation institutions.

Preservation institution of Holotype	Country	No. of species	Percentage (%)
IZAS	China	911	25.18
NWAFU	China	590	16.31
NKU	China	534	14.76
CAU	China	409	11.3
GU	China	387	10.7
AAU	China	210	5.8
TNHM	China	120	3.32
NJAU	China	101	2.79
IPPE	China	71	1.96
IMNU	China	53	1.46
SAU	China	38	1.05
SYSU	China	37	1.02
KZAS	China	13	0.36
BFUC	China	13	0.36
NWIPB	China	12	0.33
ZJAFU	China	10	0.28
SDAU	China	8	0.22
JXAU	China	8	0.22
BMNH	UK	7	0.19
IAPQ	China	6	0.17
NMNS	China	5	0.14
ZIN	Russia	4	0.11
MSTC	China	4	0.11
ICSCU	China	4	0.11
SAAS	China	4	0.11
BJMNH	China	4	0.11
FAFU	China	3	0.08
SNU	China	3	0.08
AHUT	China	3	0.08
INRSC	China	3	0.08
ISCK	China	3	0.08
SWU	China	2	0.06
RIRI	China	2	0.06
HBAU	China	2	0.06
ZISP	Russia	2	0.06
ANIC	Australia	2	0.06
SU	China	1	0.03
SCAU	China	1	0.03
AHFP	China	1	0.03
HBAF	China	1	0.03
FDYN	China	1	0.03
FJAS	China	1	0.03
GZAS	China	1	0.03
NMCU	UK	1	0.03
HSFB	China	1	0.03
HBMN	China	1	0.03
HEBNU	China	1	0.03
GZAF	China	1	0.03
MNHN	France	1	0.03
NEFU	China	1	0.03
SCU	China	1	0.03
YZU	China	1	0.03
PCPC	China	1	0.03
NHMW	Austria	1	0.03
NCHU	China	1	0.03
TARI	China	1	0.03
CATAS	China	1	0.03
YLNU	China	1	0.03
PPLS	China	1	0.03
IMAU	China	1	0.03
PSQS	China	1	0.03
NCSU	USA	1	0.03
MIZ	Poland	1	0.03
IAEAS	China	1	0.03
IRSNB	Belgium	1	0.03
HUS	Japan	1	0.03
Total		3618	100

**Table 3.** Abbreviations and full names of the preservation institutions of type specimens for Hemipteran insects in China.

**Abbreviation**	**Country**	**The full name of the preservation institution**
AAU	China	Anhui Agricultural University, Hefei, Anhui, China
AFNX	China	Ningxia Academy of Agriculture and Forestry Sciences, Ningxia, China
AHFP	China	Anhui Forest Pest Control Station, Hefei, Anhui, China
AHUT	China	Anhui University of Technology, Maanshan, Anhui, China
ANIC	Australia	Australian National Insect Collection, CSIRO, Canberra, Australia
BFUC	China	Insect Collection, the Department of Forestry Protection, Beijing Forestry University, Beijing, China
BJMNH	China	Beijing Museum of Nature History, Beijing, China
BMHU	USA	Bhopal Museum, Hawaii, USA
BMNH	UK	Natural History Museum, London, UK
BPBM	USA	Bernice P. Bishop Museum, Honolulu, Hawaii, USA
CATAS	China	Chinese Academy of Tropical Agricultural Sciences Environment and Plant Protection Institute, Haikou, Hainan, China
CAU	China	Department of Entomology, China Agricultural University, Beijing, China
CDFA	USA	California Department of Food and Agriculture, Sacramento, CA, USA
CEHI	Austria	Collection Ernst Heiss, Tiroler Landesmuseum, Innsbruck, Austria
CLHC	China	Collection of Li He, Chengdu, China
FAFU	China	College of Plant Protection, Fujian Agriculture Forestry University, Fuzhou, Fujian, China
FAHN	China	Hunan Academy of Forestry, Changsha, Hunan, China
FDYN	China	The Forestry Department of Yunnan Province, Kunming, Yunnan, China
FJAS	China	Institute of Entomology, Fujian Agriculture of Science, Fuzhou, Fujian, China
GU	China	Guizhou University, Guiyang, Guizhou, China
GXAS	China	Biological Research Laboratory, Guangxi Academy of Sciences, Nanning, Guangxi, China
GZAF	China	Guizhou Academy of Forestry, Guiyang, Guizhou, China
GZAS	China	Guizhou Academy of Sciences, Guiyang, Guizhou, China
HBAF	China	Shijiazhuang Orchard Research Institute, Hebei Academy of Agriculture and Forestry Sciences, Shijiazhuang, Hebei, China
HBAU	China	Agricultural University of Huabei, Baoding, Hebei, China
HBMN	China	Museum of Natural History, Harbin, Heilongjiang, China
HBU	China	Hebei University, Baoding, China
HEBNU	China	Hebei Normal University, Shijiazhuang, Hebei, China
HSFB	China	Huangshan City Forestry Bureau of Anhui Province, Huangshan, Anhui, China
HUS	Japan	Laboratory of Systematic Entomology, Hokkaido University, Sapporo, Japan
IAEAS	China	Shenyang Institute of Applied Ecology, Chinese Academy of Sciences, Shenyang, Liaoning, China
IAPQ	China	Institute of Animal and Plant Quarantine, Yunnan, China
ICSCU	China	Insect Collection, Gold Mantis School of Architecture and Urban Environment, Soochow University, Suzhou, Jiangsu, China
IMAU	China	Inner Mongolia College of Forestry, Hohhot, Inner Mongolia, China
IMNU	China	Inner Mongolia Normal University, Hohhot, Inner Mongolia, China
INRSC	China	Institute for Natural Resources in Sichuan, Chengdu, Sichuan, China
IPPE	China	Shanghai Institute of Biological Sciences, Chinese Academy of Sciences, Shanghai, China
IRSNB	Belgium	The Institut royal des Sciences naturelles de Belgique, Bruxelles, Belgium
IRTUA	Japan	Laboratory of Insect Resources, Faculty of Agriculture, Tokyo University of Agriculture, Atsugi, Japan
ISCK	China	The Institute of South China Karst, Guizhou Normal University, Guiyang, Guizhou, China
IZAS	China	Institute of Zoology, Chinese Academy of Sciences, Beijing, China
JXAU	China	Jiangxi Agricultural University, Nanchang, Jiangxi, China
KZAS	China	Kunming Institute of Zoology, Chinese Academy of Sciences, Kunming, Yunnan, China
MIZ	Poland	Museum and Institute of Zoology PAS, Warsaw, Poland
MNHN	France	The Muséum national d’Histoire naturelle, Paris, France
MNHU	Germany	Museum fuÈr Naturkunde der Humboldt-UniversitaÈt, Berlin, Germany
MSTC	China	Anhui Maanshan Science and Technology Commission, Maanshan, Anhui, China
NCHU	China	National Chung Hsing University, Taichung, Taiwan, China
NCSU	USA	North Carolina State University Insect Collection, Raleigh, North Carolina, USA
NCTN	Netherlands	Nieser and Chen Collection, Tiel, The Netherlands
NEFU	China	Institute of Entomology, College of Forestry, Northeast Forestry University, Harbin, Heilongjiang, China
NHMW	Austria	Naturehistorisches Museum in Wien, Wien, Austria
NJAU	China	Nanjing Agricultural University, Nanjing, Jiangsu, China
NKU	China	Institute of Entomology, College of Life Sciences, Nankai University, Tianjin, China
NMCU	UK	National Museum of Cardiff, UK
NMNS	China	National Museum of Natural Science, Taichung, Taiwan, China
NWAFU	China	Entomological Museum of Northwest A&F University, Yangling, Shaanxi, China
NWIPB	China	Northwest Institute of Plateau Biology, Chinese Academy of Sciences, Xining, Qinghai, China
PCPC	China	Private Collection of Pingping Chen, Beijing, China
PPLS	China	Plant Protection Laboratory of Shenyang Garden Science Institute, Shenyang, Liaoning, China
PSQS	China	The Plant Protection Station of Qiannan State, Guizhou, China
RIRI	China	Research Institute of Resource Insects, Chinese Academy of Forestry, Kunming, Yunnan, China
RMNUS	Singapore	Zoological Reference Collection, Raffles Museum of Biodiversity Research, National University of Singapore, Singapore
SAAS	China	College of Plant protection, Sichuan Academy of Agricultural Sciences, Chengdu, Sichuan, China
SAU	China	Insect Collection of Shanxi Agricultural University, Taigu, Shanxi, China
SCAU	China	South China Agricultural University, Guangzhou, Guangdong, China
SCU	China	Animal Herbarium, College of Life Sciences, Sichuan University, Chengdu, Sichuan, China
SDAU	China	The Research Center of Scale Insects, Shandong Agricultural University, Tai’an, Shandong, China
SHBG	China	Shanghai Botanical Garden, Shanghai, China
SNU	China	Shaanxi Normal University, Xi’an, Shanxi , China
SU	China	Department of horticultural science and technology of Soochow University, Suzhou, Jiangsu, China
SWU	China	Southwest University, Chongqing, China
SYSU	China	Sun Yat-sen University, Guangzhou, Guangdong, China
TARI	China	Taiwan Agricultural Research Institute, Taichung, Taiwan, China
TNHM	China	Tianjin Natural History Museum, Tianjin, China
USNM	USA	National Museum of Natural History, Washington D.C., USA
XIEG	China	Xinjiang Institute of Ecology and Geography, Chinese Academy of Sciences, China
YIB	Russia	Institute for Biological Problems of Cryolithozone RAS, Yakutsk, Russia
YLNU	China	College of Life Sciences, Yulin Normal University, Yulin, Guangxi ,China
YZU	China	Insect Collection of Yangzhou University, Yangzhou, Jiangsu, China
ZIN	Russia	Russian Academy of Sciences, Zoological Institute, St.Petersburg, Russia
ZISP	Russia	The Zoological Institute RAS, St. Petersburg, Russia
ZJAFU	China	The Research Center of Scale Insects, Zhejiang A&F University, Lin’an, Zhejiang, China
ZSI	India	Zoological Survey of India
